# The Utility of Scatter Feeding as Enrichment: Do Broiler Chickens Engage with Scatter–Fed Items?

**DOI:** 10.3390/ani11123478

**Published:** 2021-12-07

**Authors:** Brittany Wood, Christina Rufener, Maja M. Makagon, Richard A. Blatchford

**Affiliations:** Center for Animal Welfare, Department of Animal Science, University of California, Davis, 1 Shields Avenue, Davis, CA 95616-8531, USA; bwood@ucdavis.edu (B.W.); cbrufener@ucdavis.edu (C.R.); mmakagon@ucdavis.edu (M.M.M.)

**Keywords:** broiler, environmental enrichment, scatter feed, foraging, engagements

## Abstract

**Simple Summary:**

In recent years, there has been increasing interest in providing an enriched environment to broiler chickens. Indeed, many welfare certification companies encourage or require enrichment to be provided. Most of these companies suggest the use of scatter feeding as enrichment material, though there is little scientific evidence to support the implementation of a scatter feeding program. One of the potential benefits of scatter feeding programs may be an observed increase in foraging behavior, and hence overall activity of the birds. This study aimed to understand the impact of scatter feeding on the foraging behavior of broilers. Six groups of broilers were provided with either dried mealworms, whole wheat, shredded cabbage, alfalfa pellets, wood shavings, or no scatter feeding. To maintain the birds’ interest in the enrichment, feed items were only scattered on the first three days of each week. Foraging and feeding behavior were observed via video for one-hour periods immediately after scattering, 2 h later, and 6 h later. Immediately following the scattering of feed items, broilers in all groups showed an increase in foraging, though this was most pronounced in the dried mealworm group. Foraging behavior decreased with age for all groups. The mealworm group also fed less during hour one compared to the later hours. These results did not provide evidence that scatter feeding encourages foraging behavior, except for a short-term effect of a high value food item. Therefore, future studies should examine the feed item and delivery in more detail.

**Abstract:**

In recent years, welfare certification companies have encouraged the use of scatter feeding as enrichment material, though there is little scientific evidence to support a scatter feeding program. This study aimed to understand the impact of scatter feeding on the foraging behavior of broilers. One hundred eighty Ross 308 chicks were allocated into six treatment groups (six replicates/treatment). Broilers were scatter fed dried mealworms, whole wheat, shredded cabbage, alfalfa pellets, wood shavings, or no scatter feeding, respectively. Enrichment was provided on the first three days of each week. Total foraging, active foraging, and feeding were observed for one-hour periods immediately after scattering, 2 h later, and 6 h later. In all groups, broilers increased both total (*p* = 0.001) and active (*p* = 0.001) foraging, though this was most pronounced in the dried mealworm group. Across all groups, active foraging decreased with age (*p* = 0.001). The mealworm group also showed a corresponding decrease in feeding during hour one compared to the later hours (*p* = 0.001). These results did not provide evidence that scatter feeding encourages foraging behavior, except for a short-term effect of a high value feed item. This finding suggests that the item scattered and the delivery method should be studied further.

## 1. Introduction

Environmental enrichments are modifications of the environment that aim to promote the performance of normal behaviors and/or animal health. In broiler chicken production, environmental enrichment is often provided with the goal of increasing overall activity, and preventing the development of lameness, hock burns, and breast blisters [1]. In the US, third-party animal welfare certification programs typically require that broiler chickens are housed with enrichment materials ex. [2,3]. Scatter feeding is often listed among the recommended forms of enrichment. Its application is based on the hypothesis that this practice will promote foraging behavior and overall activity. However, studies investigating foraging in broilers have failed to find a definitive relationship between scattering of feed stuff and broiler activity levels. The scattering of whole wheat, for example, did not increase broiler activity as reported in several recent studies [4,5,6]. Other feed items, such as mealworms, have resulted in only short-term increases in foraging activity [6]. The current study aims to add to the existing body of literature by evaluating how feeding and foraging behavior change immediately following the scattering of feed and on days and at times when feed stuff is not scattered, as well as assessing which feed items have the most pronounced impact on broiler behavior.

The idea that the scattering of whole grains or other feed items can be used as a form of environmental enrichment for broilers is grounded in the assumption that foraging behavior is an important part of the normal behavioral repertoire of broilers. For example, a welfare certification organization [2] states that “if chickens are provided with edible material contained in their litter, they will be actively engaged in foraging behavior for extended periods,” while another one [3] lists foraging as an example of a natural behavior that should be encouraged. However, what constitutes a normal behavior (i.e., behavior that is important to the animal in a particular environmental context) can be modified by selective breeding and environmental conditions. It has been suggested that, along with selection for fast growth, the broiler behavioral repertoire has shifted towards the performance of behaviors that allow the birds to conserve their energy [7]. For example, while red junglefowl allocate a large proportion of their day to foraging-related activities such as pecking the ground or scratching the litter [8], modern-day broilers have been shown to spend over 60% of the day inactive and less than 4% of daylight hours foraging [9,10]. Moreover, while red junglefowl will search for feed even when they are provided with a “free” feed option [11], broilers do not show this tendency [7]. If scattering of feed is to be recommended as a form of environmental enrichment for broilers, further investigation into whether scattering of feed effectively promotes foraging behavior is warranted. The overall goal of this study is to evaluate whether scatter feeding is a viable form of enrichment for broiler chickens. Specifically, we assessed whether broiler chickens would forage for scattered feed items when provided, and whether increased activity would be observed on days when scattered feed was not offered. We further investigated whether broilers would engage with some feed items compared to others.

## 2. Materials and Methods

The study was conducted over six weeks between June and July 2018 at the Hopkins Avian Facility, University of California, Davis, with approval from the UC Davis Institutional Animal Care and Use Committee (Protocol #20212, approved November 2017).

### 2.1. Animals and Housing

One hundred ninety-five mixed-sex day-old Ross 308 chicks were reared on wood shavings. The chicks were acquired from a commercial hatchery, and individually marked with food coloring on day one of age. Chicks were colored with 5 colors, grouped into 5 pens by color and brooded for five days under ceramic heating bulbs in six identical pens (3.05 m × 1.52 m). A sixth group of 15 extra chicks were not colored. During brooding, chicks had ad libitum access to water and commercially supplied starter feed delivered in a standard 3.5-gal (13.25 L) waterer and 30 lb (13.61 kg) round feeder, respectively. For the first three days, chicks received 23 L:1 D hours light: dark. Daylight hours were subsequently reduced to 20 L:4 D. On day six of age, chicks were placed into 36 pens (3.05 m × 1.52 m; in groups of 5–6. The groups were composed of one chick from each of the colored brooding pens. Unmarked (extra) chicks were distributed as evenly as possible across the pens. An opaque tarp was hung across pen partitions to prevent chicks in one group from seeing those in adjacent pens. Ad libitum access to water and commercially supplied starter (1 week), grower (2 weeks), and finisher (2 weeks) diets continued to be provided. Researchers and staff entered the barn only to conduct daily wellness and equipment checks, and to clean and refill feeders/waterers.

### 2.2. Research Treatments

Each of the thirty-six pens was assigned to one of six treatments (six replicates/treatment): (1) dried mealworms (MW), (2) whole wheat (WW), (3) cabbage (CA), (4) alfalfa pellets (AP), (5) wood shavings (SH), and (6) feed-only/no-scattering control (Control). Treatments were assigned in blocks to ensure that they were uniformly distributed across the barn. Enrichments were scattered evenly across the pen floor on the first three days of each week between 10:00 and 11:00 h. Birds in the MW, CA, AP, and SH treatment pens received a half cup (118.3 mL) of enrichment. Due to the difference in grain size (grain units), only ¼ cup (59.15 mL) of WW was used. The Control group served as a non-scatter-feeding control. Specifically, we tested how the scattering of whole wheat, cabbage, alfalfa, dried mealworms, broiler feed, and shavings impacted the feeding and foraging activity of broiler chickens. Due to the fact that the proposed benefits of scatter feeding are linked to increased activity and load placed on the legs, we further differentiated between active and inactive foraging, where active foraging was performed while the bird was standing up or walking. The whole wheat and cabbage treatment were selected for evaluation as they are currently listed as effective forms of enrichment by one or more broiler welfare assurance programs ex. [2,3]. Alfalfa pellets were included because they are easily accessible to producers; therefore, they were selected due to their potential to serve as a practical enrichment. Wood shavings were used to test the impact of the act of scattering (a non-nutritive resource) on broiler behavior. The dried mealworm treatment was included as a positive control. Mealworms are considered a high-value feed item, are commonly used as a feed reward in research [12,13,14], and have been shown to have some impact on foraging behavior in previous studies [6]. The feed-only treatment served as the negative control.

### 2.3. Behavioral Observations

A DVR furnished with GeoVision-1480 video surveillance system software and connected to 36 video cameras (Clinton, Model CE-VF540, Clinton Electronics, Loves Park, IL, USA; 1 video camera per pen) recording chick behavior within the entire floor area of each pen. Video recorded on the first and fourth day of weeks 2 and 4, and the first day of week 6, was subsequently analyzed. The first day of each week (ON day) represented the first day of enrichment delivery, while day four (OFF day) represented the first day within the week when enrichment was not scattered. Three one-hour observation periods were monitored on each focal day. The exact start time for the observation was established independently for each pen each week. Scattering occurred between 10:00 and 11:00. During ON days, behavioral observations commenced immediately after enrichment was scattered and the researcher moved completely out of the video frame (H1). The same observation start time was used for the OFF day observations within a given pen each week. Control was not provided with enrichment. The observations for those 5 pens began 30 min after the adjacent pens received enrichment. The remaining daily observation periods took place two hours after the last set of H1 observations, at approximately 13:00–14:00 (H2) and 17:00–18:00 (H3).

Behavioral data were collected using the 1-0 scan sampling strategy. Observers reviewed the first of every five minutes of video and recorded whether each of the color-marked birds participated in feeding from the feeder or in foraging behavior during that minute. Chicks were assumed to be feeding from the feeder if they were observed pecking within the feeder trough. Chicks observed pecking at the ground or raking their beaks across or scratching the wood shavings were assumed to be foraging. We considered chicks to be “foraging active” if they were standing or walking while foraging, and “foraging inactive” if they were sitting or lying down. In total, 12 observations were recorded per hour, per chick, and per pen.

Five observers assisted with data collection. Before engaging in data collection, the observers were trained on the data collection protocol, and their inter-rater reliability was evaluated against that of the lead researcher (B.W.; at least 90% agreement was required). Inter-rater reliability was assessed based on a review of two hours of video footage (one hour recorded in the morning and one in the evening). Additional two-hour video clips taken from a variety of cameras and representing a variety of chick ages were assigned to all of the observers over the course of the study to ensure that reliability remained consistently high.

### 2.4. Data Processing

For each focal hour of behavioral observations, we calculated the percentage of observations (out of 12 possible) during which each individual broiler chicken engaged in “foraging active”, “foraging inactive”, and “feeding”. The proportion of observations during which each individual engaged in any type of foraging (“foraging active” + “foraging inactive”) was also calculated (“foraging total”). An initial visual comparison of means revealed that means were similar across the observations when no scattering was provided, i.e., during H2 and H3 on ON days, and all observations on OFF days. Therefore, the analysis included data from ON days only. Observations during H2 and H3 were combined to allow for comparison between observations immediately after scattering (H1) vs. later in the day (H2 and H3). Based on the visual comparison of means, we combined treatments to Control, Other (WW, CA, AP, SH; all treatments where scattering was provided except for MW), and Mealworms (MW).

### 2.5. Statistical Analysis

Statistical analysis was completed in R 3.4.2 (R Core Team, 2017) using linear mixed-effect models (LMER) with package ‘lme4’ [15]. A graphical analysis was used to confirm homoscedasticity of explanatory variables and normality of residuals. Outcome variables were transformed as needed. The final model was obtained with a stepwise backward reduction and a *p*-value > 0.05 as a criterion of exclusion using parametric bootstrap tests (package ‘pbkrtest’ [16]). The ‘effects’ package [17] was used to calculate model estimates.

Outcome variables were the percent of observations spent foraging total, the percent of observations spent foraging actively (square root transformed), and the percent of observations spent feeding. Fixed effects were age (factor with 3 levels: 2, 4, 6 weeks of age), treatment (factor with 3 levels: Control, Other, Mealworms), hour (factor with 2 levels: H1, H2 and H3), and the interaction of hour and treatment. To account for repeated measures and pseudo-replication, as well as pen-to-pen and individual-to-pen variation, hour nested in week nested in individual nested in pen was included as a random effect.

## 3. Results

### 3.1. Foraging Total

**Figure 1:**
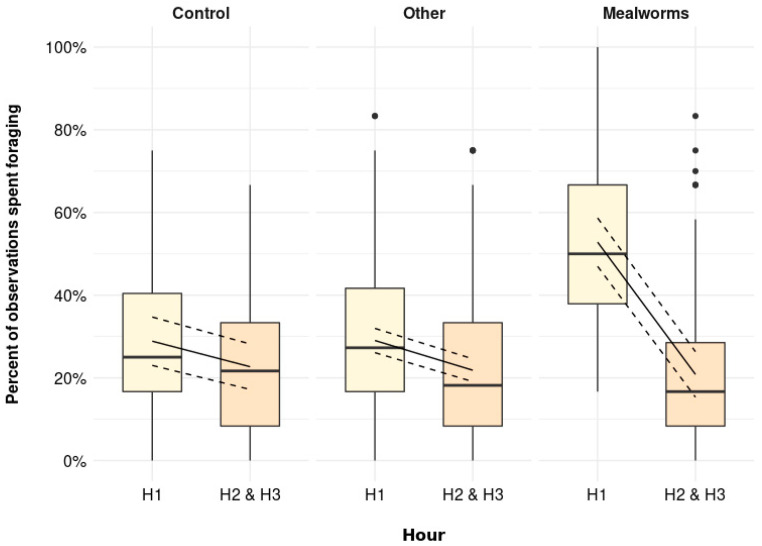
The percentage of observations spent foraging (including foraging active and foraging inactive) immediately after scattering was provided (H1) and later in the day (H2 and H3) for Control (no scattering), Other (scattering of shavings, whole wheat, alfalfa, or cabbage), and Mealworms (*p* = 0.001). Boxplots show medians, lower, and upper interquartile range of raw data. Whiskers indicate 1.5 times the interquartile range. Solid lines represent estimated means and dashed lines represent 95 % confidence intervals.

The percentage of observations spent foraging was higher immediately after the scattering was provided (H1) than later in the day (H2 and H3) in all treatments, but this pattern was most pronounced in the MW treatment (Figure 1, *p* = 0.001). More specifically, the estimated means [95% confidence interval] were similar during H2 and H3 irrespective of treatments (Control: 22.7 [17.2, 28.2] %, Other: 21.8 [19.1, 24.6] %, MW: 20.8 [15.3, 26.3] %). Whereas the percent of observations spent foraging during H1 was increased by only few percent in Control and Other birds (Control: 28.9 [23.0, 34.7] %, Other: 29.0 [26.1, 32.0] %), MW birds were foraging during 52.8 [47.0, 58.7] % of the observations in H1.

**Figure 2:**
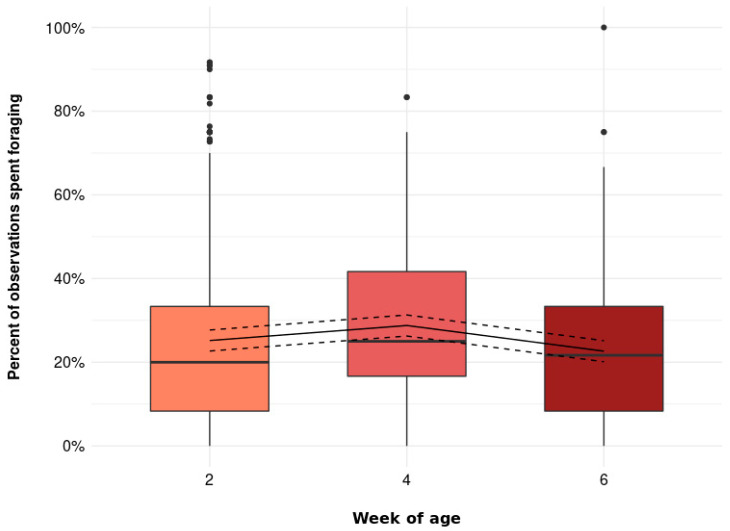
The percent of observations spent foraging (including foraging active and foraging inactive) at 2, 4, and 6 weeks of age (*p* = 0.001). Boxplots show medians, lower, and upper interquartile range of raw data. Whiskers indicate 1.5 times the interquartile range. Solid lines represent estimated means and dashed lines represent 95% confidence intervals.

The percent of observations spent foraging was further affected by age (Figure 2, *p* = 0.001), though the effect was small (2 weeks of age: 25.2 [22.7, 27.7] %, 4 weeks of age: 28.8 [26.3, 31.3] %, 6 weeks of age: 22.6 [20.1, 25.1] %).

### 3.2. Foraging Active

**Figure 3:**
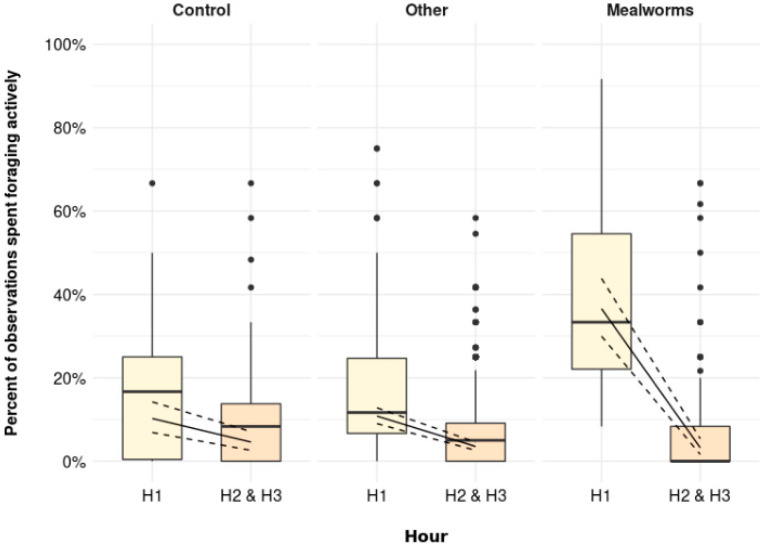
The percentage of observations spent foraging actively immediately after scattering was provided (H1) and later in the day (H2 and H3) for Control (no scattering), Other (scattering of shavings, whole wheat, alfalfa, or cabbage), and Mealworms (*p* = 0.001). Boxplots show medians, lower, and upper interquartile range of raw data. Whiskers indicate 1.5 times the interquartile range. Solid lines represent estimated means and dashed lines represent 95% confidence intervals.

Similar to the percentage of observations spent foraging in total, the percentage of observations spent foraging actively was higher immediately after the scattering was provided (H1) than later in the day (H2 and H3) in all treatments. Again, this pattern was most pronounced in the MW treatment (Figure 3, *p* = 0.001). Whereas birds were foraging actively to a low percent irrespective of treatment during H2 and H3 (Control: 4.6 [2.6, 7.1] %, Other: 3.5 [2.6, 4.6] %, MW: 3.2 [1.6, 5.4] %), the percent of observations spent foraging actively was increased by a few percent during H1 in Control (10.2 [6.9, 14.2] %) and Other (10.8 [9.0, 12.8] %). MW birds, on the other hand, spent 36.6 [30.0, 43.9] % of the observations during H1 foraging actively.

**Figure 4:**
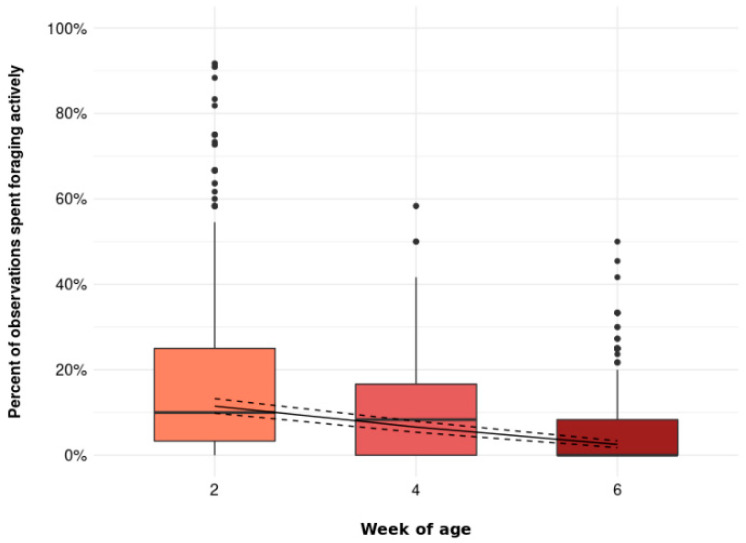
The percentage of observations spent foraging actively at 2, 4, and 6 weeks of age (*p* = 0.001). Boxplots show medians, lower, and upper interquartile range of raw data. Whiskers indicate 1.5 times the interquartile range. Solid lines represent estimated means and dashed lines represent 95% confidence intervals.

In addition, the percentage of observations spent foraging actively decreased with increasing age (Figure 4, *p* = 0.001). Birds were foraging actively in 11.5 [9.9, 13.2] % of the observations at 2 weeks of age, in 6.6 [5.4, 8.0] % of the observations at 4 weeks of age, and in 2.5 [1.8, 3.4] %) of the observations at 6 weeks of age.

### 3.3. Feeding

**Figure 5:**
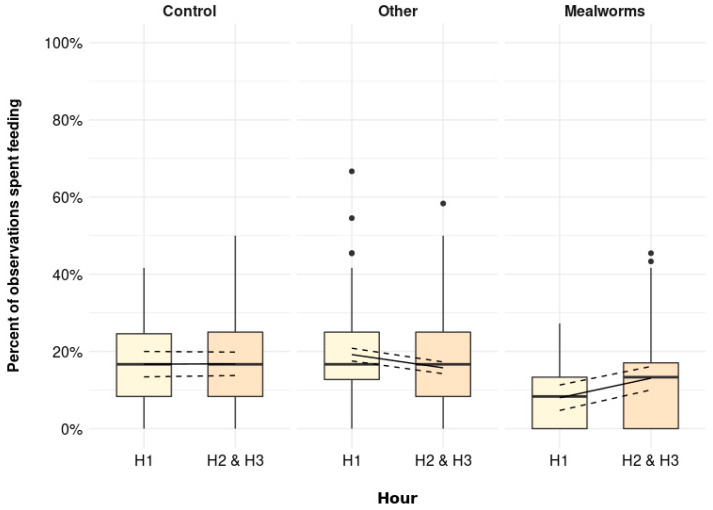
The percentage of observations spent feeding at the feeder immediately after scattering was provided (H1) and later in the day (H2 and H3) for Control (no scattering), Other (scattering of shavings, whole wheat, alfalfa, or cabbage), and Mealworms (*p* = 0.001). Boxplots show medians, lower, and upper interquartile range of raw data. Whiskers indicate 1.5 times the interquartile range. Solid lines represent estimated means and dashed lines represent 95% confidence intervals.

The percentage of observations spent feeding at the feeder was similar across all observations, but birds in the Mealworms treatment were observed feeding less during H1 (Figure 5, *p* = 0.001). More specifically, Mealworm birds were feeding in 8 [4.7, 11.3] % of the observations during H1, which was lower compared to all other observations (H1 Control: 16.7 [13.4, 20.0] %, H1 Other: 19.2 [17.5, 20.8], H2 and H3 control: 16.9 [13.8, 19.8] %, H2 and H3 Other: 15.7 [14.2, 17.3], H2 and H3 Mealworms: 13.1 [10.0, 16.1] %).

**Figure 6:**
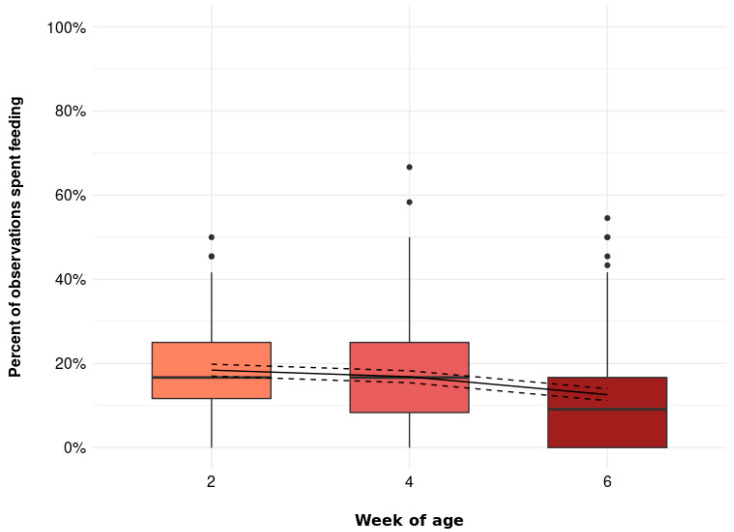
The percentage of observations spent feeding at the feeder at 2, 4, and 6 weeks of age (*p* = 0.001). Boxplots show medians, lower, and upper interquartile range of raw data. Whiskers indicate 1.5 times the interquartile range. Solid lines represent estimated means and dashed lines represent 95% confidence intervals.

The percentage of observations spent feeding at the feeder was further affected by age (Figure 6, *p* = 0.001), though the effect was small (2 weeks of age: 18.4 [17.0, 19.8] %, 4 weeks of age: 16.8 [15.4, 18.3] %, 6 weeks of age: 12.6 [11.2, 14.0] %).

## 4. Discussion

Scattering of feed with the goal of promoting foraging behavior and overall activity is a recommended form of broiler enrichment. Several previous studies have, however, failed to find a relationship between the scattering of feed and foraging and/or overall activity [4,5,6]. When noted, impacts of scatter feeding on broiler behavior have been associated with the scattering of high-value feed items, such as mealworms, but have had short-term impacts on broiler behavior [6].

Across all treatments, foraging activity was higher during H1 than subsequently in the day. In line with previous work, different feed items had different impacts on broiler behavior [4,5,6]. Along with type, the presence of the feed item also contributes to observed increases in foraging activity, as indicated by the fact that foraging activity increased only during H1 and only on days when scatter feed was delivered. However, as is consistent with other studies [4,9,10], broiler activity (in the current study, foraging) still suffered a reduction as age increased, even in treatments that initially stimulated foraging. This is a reoccurring issue with birds selected for fast growth, as it is difficult to bypass the confounding effect of age (and size) on overall locomotive activity levels.

In the current study, mealworms stimulated the most total and active foraging activity. However, the increase in foraging activity in the mealworm treatment only increased during H1 of the observations. Pichova et al. [6] investigated the effects of whole wheat, wood shavings, and mealworms on activity levels in broilers. They also found that scattering mealworms on the litter once per day encouraged activity such as litter pecking and scratching, and that the change in behavior only occurred immediately after mealworm delivery. In both studies, enrichment items were only scattered once per day, and this may have had an impact on the amount of foraging behavior observed. It could be that the significance of the items scattered decreased over time, as the items were consumed, and their presence in the environment decreased. Pichova et al. [6] also observed motivational differences in litter directed behavior for scattered feed items on litter. This may be attributable to the fact that, as the visual stimulation of the items decreased, the motivation of the birds to forage also decreased.

The increase in foraging among mealworm-fed birds during H1 was associated with a reduction in the percentage of observations of birds feeding. This is not surprising, as these behaviors are mutually exclusive. Overall, the percentage of observations that the broilers spent feeding was similar across treatments, suggesting that the amount of scattered feed items did not interfere with feed intake. Although there was a very small effect, observed feeding behavior decreased with age, as also shown by Alvino et al. [10]. The small change in feeding behavior observed may be due to sampling time. In the current study, behavior was observed over three one-hour periods. Feeding behavior has been shown to have a strong circadian rhythm [9,18,19], and it could be stated that the chosen observation times were not optimal for measuring feeding behavior.

The results from the current study suggest that there is little effect of scatter feeding enrichments on stimulating broilers to perform foraging activity. However, Pichova et al. [6] suggested future research should focus on developing feed items that are highly attractive and distributed in ways that would increase broiler activity. For instance, the current study used whole wheat, which is a frequently recommended scatter enrichment item, and showed no effect on activity, which is in line with several studies. However, there is often little consideration given to the environments in which studies are conducted. It may be that experimental scale studies, small numbers of animals, small spaces, etc., may have an effect on the behavior of the broilers, which may differ under large scale commercial conditions. In the current study, scattering of items was carried out in close proximity to the feeders, where feed was available ad libitum. Therefore, the broilers may have been less motivated to seek out scattered items through foraging. Jordan et al. [5] found that when feeders were removed, broilers were more likely to be active when pelleted feed was scattered. Although they reported no increased activity when whole wheat was scattered, they did not remove feeders for this treatment. Therefore, future scatter feeding studies should test items under commercial conditions where broilers are likely to have times that they are not near feeder lines [20], and this may increase the likelihood of foraging even for items shown experimentally not to be as effective. Recently, Ferreira et al. [14] also found individual differences in the display of foraging behavior by broiler chickens, which may have implications on measuring this behavior when comparing treatments across the group level. Future studies should consider investigating and comparing results between individuals and groups.

## 5. Conclusions

As indicated, this study did not provide evidence that scatter feeding, as currently recommended in welfare certification guidelines, promotes long term foraging. In this study, foraging behavior was only encouraged in broilers scatter fed with mealworms, a well-known, high-value feed item. These results coincide with previous studies using mealworms as a source of environmental enrichment. Although broilers were stimulated to forage, this occurred only on the days when enrichment was provided (ON days) and only directly after distribution. Despite not being able to modify foraging behavior in the long term, there is a chance that, with the appropriate enrichment, delivery method, and schedule, broiler activity can be increased. It may also be of interest to look at the entire behavioral repertoire, instead of only foraging, as scatter feeding may have effects outside of the measures in the current study.

It is recommended that broilers are provided with high-value feed items that will motivate foraging behavior and general activity. Suggested environmental enrichment that promotes locomotor activity, such as mealworms, bales of straw, feed pellets, and various light intensities, may be used in order to increase activity. Enrichment has been found to effectively induce broiler activity when used in combinations. For example, straw bales and light intensity show promising results in promoting activity, which can lead to a reduction in lameness [21]. As enrichment stimulated foraging primarily within the first hour, in the future, broilers should probably receive enrichment more frequently and at various times of the day to initiate and prolong foraging activity. The use of various or a combination of enrichments throughout the development period may be worth exploring to keep enrichment novel and engaging, encourage locomotion, increase foraging behavior, and possibly contribute to improved leg health.

## Data Availability

The raw data supporting the conclusions of this article will be made available by the authors, without undue reservation.
